# Supplementary material for: An Innovative Structural Rearrangement in Imine Palladacycle Metaloligand Chemistry: From Single-Nuclear to Double-Nuclear Pseudo-Pentacoordinated Complexes
Source: Molecules. 2023 Mar 2;28(5):2328. doi: 10.3390/molecules28052328 (PMC10005383; doi:10.3390/molecules28052328)
Supplement: Supplementary file 1 [file molecules-28-02328-s001.zip › molecules-2222977-supplementary.pdf]

Tables S1-S3 are for the crystal structure for 10 obtained from compound 3a

Table S1 Crystal data and structure refinement for 10 (from 3a)

Identification code	21VAA016
Empirical formula	$C_{88}H_{92}F_{12}N_2P_8Pd_2$
Formula weight	1866.326
Temperature/K	100.00
Crystal system	monoclinic
Space group	$P2_1/n$
a/Å	11.6509(4)
b/Å	22.6116(9)
c/Å	15.5974(7)
α/°	90
β/°	96.339(1)
γ/°	90
Volume/Å^3	4083.9(3)
Z	2
ρ_{calc}g/cm^3	1.518
μ/mm^{-1}	0.672
F(000)	1906.8
Crystal size/mm^3	0.11 × 0.07 × 0.03
Radiation	Mo Kα (λ = 0.71073)
2Θ range for data collection/°	4.52 to 52.74
Index ranges	$-14 \le h \le 12$, $-28 \le k \le 28$, $-19 \le l \le 19$
Reflections collected	86534
Independent reflections	8351 [R_{int} = 0.0539, R_{sigma} = 0.0256]
Data/restraints/parameters	8351/0/505
Goodness-of-fit on F^2	1.050
Final R indexes [I>=2σ (I)]	R_1 = 0.0384, wR_2 = 0.0903
Final R indexes [all data]	R_1 = 0.0460, wR_2 = 0.0945
Largest diff. peak/hole / e Å^{-3}	1.14/-0.65

Table S2 Bond Lengths for 10 (from 3a).

Atom	Atom	Length/Å	Atom	Atom	Length/Å
Pd1	P1	2.3126(8)	C39	C44	1.397(5)
Pd1	P3	2.3108(8)	C39	C40	1.384(5)
Pd1	P2	2.3375(7)	C42	C43	1.370(6)
Pd1	N1	2.296(2)	C17	C22	1.396(5)
Pd1	C1	2.042(3)	C17	C18	1.385(5)
P1	C17	1.816(3)	C11	C12	1.379(5)
P1	C11	1.813(3)	C11	C16	1.400(5)
P1	C23	1.843(3)	C28	C27	1.362(6)
P3	C39	1.819(3)	C28	C29	1.393(6)
P3	C32	1.840(3)	C22	C21	1.388(6)
P3	C33	1.816(3)	C44	C43	1.388(5)
P2	C25	1.824(3)	C32	C31	1.523(4)
P2	C31	1.838(3)	C5	C10	1.523(5)
P2	C24	1.839(3)	C5	C6	1.516(5)
P4	F2	1.594(2)	C9	C10	1.538(5)
P4	F1	1.588(2)	C9	C8	1.518(6)
P4	F4	1.601(2)	C34	C33	1.397(5)
P4	F6	1.578(2)	C34	C35	1.391(5)
P4	F3	1.594(3)	C33	C38	1.388(5)
P4	F5	1.590(3)	C26	C27	1.385(5)
N1	C5	1.475(4)	C19	C18	1.400(5)
N1	C4	1.277(4)	C14	C13	1.377(6)
C41	C42	1.389(6)	C14	C15	1.376(6)
C41	C40	1.389(5)	C35	C36	1.375(6)
C1	C2	1.408(4)	C12	C13	1.388(5)
C1	C3[1]	1.387(4)	C38	C37	1.407(5)
C25	C26	1.394(4)	C30	C29	1.387(5)
C25	C30	1.397(5)	C7	C8	1.503(6)
C20	C21	1.363(6)	C7	C6	1.541(5)
C20	C19	1.371(6)	C37	C36	1.364(6)
C2	C3	1.404(4)	C23	C24	1.522(5)
C2	C4	1.454(4)	C16	C15	1.383(5)

[1]-X,1-Y,-Z

Table S3 Bond Angles for 10 (from 3a).

Atom	Atom	Atom	Angle/°	Atom	Atom	Atom	Angle/°
P3	Pd1	P1	123.08(3)	C19	C20	C21	120.9(4)
P2	Pd1	P1	84.29(3)	C3	C2	C1	121.4(3)
P2	Pd1	P3	85.05(3)	C4	C2	C1	118.3(3)
N1	Pd1	P1	119.98(7)	C4	C2	C3	120.3(3)
N1	Pd1	P3	116.60(6)	C2	C3	C1[1]	121.1(3)
N1	Pd1	P2	107.26(6)	C44	C39	P3	122.4(3)
C1	Pd1	P1	93.21(8)	C40	C39	P3	117.7(2)
C1	Pd1	P3	92.08(8)	C40	C39	C44	119.3(3)
C1	Pd1	P2	174.31(8)	C43	C42	C41	120.5(3)
C1	Pd1	N1	78.42(10)	C22	C17	P1	122.2(3)
C17	P1	Pd1	121.02(11)	C18	C17	P1	119.2(3)
C11	P1	Pd1	116.67(11)	C18	C17	C22	118.4(3)
C11	P1	C17	105.60(15)	C12	C11	P1	118.9(3)
C23	P1	Pd1	102.81(11)	C16	C11	P1	122.3(3)
C23	P1	C17	104.34(16)	C16	C11	C12	118.5(3)
C23	P1	C11	104.40(15)	C29	C28	C27	120.5(3)
C39	P3	Pd1	119.06(11)	C21	C22	C17	120.7(4)
C32	P3	Pd1	104.84(10)	C43	C44	C39	120.0(4)
C32	P3	C39	101.17(14)	C31	C32	P3	109.2(2)
C33	P3	Pd1	118.22(11)	C10	C5	N1	110.0(3)
C33	P3	C39	107.50(15)	C6	C5	N1	115.7(3)
C33	P3	C32	103.30(15)	C6	C5	C10	111.1(3)
C25	P2	Pd1	125.24(10)	C8	C9	C10	111.6(3)
C31	P2	Pd1	107.56(10)	C35	C34	C33	120.4(4)
C31	P2	C25	102.87(14)	C22	C21	C20	119.9(4)
C24	P2	Pd1	107.81(10)	C39	C40	C41	120.4(3)
C24	P2	C25	106.16(15)	C34	C33	P3	119.4(3)
C24	P2	C31	105.71(15)	C38	C33	P3	120.6(3)
F1	P4	F2	89.50(13)	C38	C33	C34	119.5(3)
F4	P4	F2	178.95(14)	C27	C26	C25	120.2(3)
F4	P4	F1	90.13(13)	C18	C19	C20	119.5(4)
F6	P4	F2	90.67(14)	C19	C18	C17	120.6(4)
F6	P4	F1	89.65(15)	C15	C14	C13	120.0(4)
F6	P4	F4	90.31(14)	C36	C35	C34	119.3(4)
F3	P4	F2	90.24(13)	C13	C12	C11	120.6(4)
F3	P4	F1	179.72(14)	C37	C38	C33	119.5(4)
F3	P4	F4	90.13(13)	C29	C30	C25	120.6(3)
F3	P4	F6	90.26(17)	C9	C10	C5	110.7(3)
F5	P4	F2	89.92(14)	C6	C7	C8	110.8(3)
F5	P4	F1	90.04(16)	C36	C37	C38	119.9(4)
F5	P4	F4	89.10(15)	C32	C31	P2	110.5(2)
F5	P4	F6	179.33(17)	C2	C4	N1	119.3(3)
F5	P4	F3	90.06(17)	C44	C43	C42	120.1(3)
C5	N1	Pd1	129.02(18)	C24	C23	P1	109.1(2)
C4	N1	Pd1	110.02(19)	C7	C8	C9	112.0(3)
C4	N1	C5	120.9(3)	C26	C27	C28	120.6(4)
C40	C41	C42	119.6(4)	C37	C36	C35	121.4(4)
C2	C1	Pd1	114.0(2)	C7	C6	C5	110.0(3)
C3[1]	C1	Pd1	128.5(2)	C12	C13	C14	120.3(4)

Table S3 Bond Angles for 10 (from 3a).

Atom	Atom	Atom	Angle/°	Atom	Atom	Atom	Angle/°
C3[1]	C1	C2	117.5(3)	C15	C16	C11	120.7(4)
C26	C25	P2	122.3(3)	C23	C24	P2	111.0(2)
C30	C25	P2	119.0(2)	C16	C15	C14	119.9(4)
C30	C25	C26	118.7(3)	C30	C29	C28	119.3(4)

[1]-X,1-Y,-Z

Tables S4-S6 are for the crystal structure for 10 obtained from compound 2a

Table S4 Crystal data and structure refinement for 10 (from 2a).

Identification code	**JM VILA 21VAA045**
Empirical formula	$C_{176}H_{184}F_{24}N_4P_{16}Pd_4$
Formula weight	3732.652
Temperature/K	100.00
Crystal system	monoclinic
Space group	$P2_1/n$
a/Å	11.6417(9)
b/Å	22.6057(17)
c/Å	15.6302(13)
α/°	90
β/°	96.218(3)
γ/°	90
Volume/Å^3	4089.2(6)
Z	1
ρ_{calc}g/cm^3	1.516
μ/mm^{-1}	0.671
F(000)	1906.8
Crystal size/mm^3	0.2 × 0.2 × 0.1
Radiation	Mo Kα (λ = 0.71073)
2Θ range for data collection/°	4.52 to 56.66
Index ranges	$-15 \le h \le 15$, $-30 \le k \le 30$, $-20 \le l \le 20$
Reflections collected	135439
Independent reflections	10155 [R_{int} = 0.0817, R_{sigma} = 0.0341]
Data/restraints/parameters	10155/0/505
Goodness-of-fit on F^2	1.075
Final R indexes [I>=2σ (I)]	R_1 = 0.0341, wR_2 = 0.0756
Final R indexes [all data]	R_1 = 0.0447, wR_2 = 0.0831
Largest diff. peak/hole / e Å^{-3}	0.81/-0.55

Table S5 Bond Lengths for JM VILA 21VAA045. Compound 10 (from 2a).

Atom	Atom	Length/Å	Atom	Atom	Length/Å
Pd1	P3	2.3147(6)	C17	C18	1.400(4)
Pd1	P2	2.3416(6)	C15	C16	1.390(4)
Pd1	P1	2.3127(6)	C28	C27	1.379(5)
Pd1	N5	2.2869(19)	C28	C29	1.389(4)
Pd1	C2	2.046(2)	C8	C9	1.513(4)
P3	C39	1.820(2)	C8	C7	1.524(4)
P3	C33	1.823(2)	C2	C3	1.407(3)
P3	C32	1.838(2)	C25	C26	1.394(3)
P2	C31	1.843(2)	C25	C30	1.402(3)
P2	C25	1.822(2)	C26	C27	1.392(4)
P2	C24	1.839(2)	C39	C40	1.386(4)
P1	C17	1.813(2)	C39	C44	1.397(4)
P1	C23	1.842(2)	C34	C33	1.393(3)
P1	C11	1.819(2)	C34	C35	1.388(4)
P4	F1	1.6011(17)	C33	C38	1.380(3)
P4	F2	1.5940(18)	C42	C41	1.374(4)
P4	F4	1.5956(19)	C42	C43	1.370(5)
P4	F5	1.5844(18)	C30	C29	1.390(4)
P4	F3	1.6080(18)	C24	C23	1.530(3)
P4	F6	1.5958(19)	C36	C35	1.376(4)
C1	C2	1.388(3)	C36	C37	1.384(4)
C1	C3[1]	1.406(3)	C4	C3	1.451(3)
C5	N5	1.475(3)	C40	C41	1.394(4)
C5	C10	1.525(3)	C13	C12	1.391(4)
C5	C6	1.525(3)	C20	C21	1.372(5)
N5	C4	1.284(3)	C20	C19	1.384(4)
C10	C9	1.537(4)	C18	C19	1.392(4)
C31	C32	1.528(3)	C6	C7	1.535(4)
C22	C17	1.393(4)	C38	C37	1.394(4)
C22	C21	1.405(4)	C11	C16	1.391(4)
C14	C15	1.386(4)	C11	C12	1.397(3)
C14	C13	1.377(4)	C44	C43	1.396(4)

[1]-X,1-Y,-Z

Table S6 Bond Angles for JM VILA 21VAA045. Compound 10 (from 2a).

Atom	Atom	Atom	Angle/°	Atom	Atom	Atom	Angle/°
P2	Pd1	P3	84.22(2)	C13	C14	C15	120.4(3)
P1	Pd1	P3	122.79(2)	C22	C17	P1	120.7(2)
P1	Pd1	P2	85.06(2)	C18	C17	P1	119.24(19)
N5	Pd1	P3	120.29(5)	C18	C17	C22	119.5(2)
N5	Pd1	P2	107.21(5)	C16	C15	C14	119.6(3)
N5	Pd1	P1	116.58(5)	C29	C28	C27	119.9(3)
C2	Pd1	P3	93.36(6)	C7	C8	C9	111.7(2)
C2	Pd1	P2	174.27(6)	C1	C2	Pd1	128.43(16)
C2	Pd1	P1	91.93(6)	C3	C2	Pd1	113.87(15)
C2	Pd1	N5	78.50(8)	C3	C2	C1	117.70(19)
C39	P3	Pd1	121.04(8)	C26	C25	P2	122.5(2)
C33	P3	Pd1	116.27(8)	C30	C25	P2	118.72(18)
C33	P3	C39	105.63(11)	C30	C25	C26	118.8(2)
C32	P3	Pd1	102.85(8)	C27	C26	C25	120.3(3)
C32	P3	C39	104.65(12)	C40	C39	P3	119.2(2)
C32	P3	C33	104.51(11)	C44	C39	P3	121.7(2)
C31	P2	Pd1	107.99(8)	C44	C39	C40	118.8(2)
C25	P2	Pd1	125.41(8)	C35	C34	C33	120.6(3)
C25	P2	C31	106.04(11)	C34	C33	P3	121.96(19)
C24	P2	Pd1	107.58(8)	C38	C33	P3	118.59(19)
C24	P2	C31	105.32(11)	C38	C33	C34	119.2(2)
C24	P2	C25	102.89(11)	C43	C42	C41	120.5(3)
C17	P1	Pd1	118.32(8)	C29	C30	C25	120.4(2)
C23	P1	Pd1	104.86(8)	C26	C27	C28	120.5(3)
C23	P1	C17	103.39(11)	C23	C24	P2	110.33(16)
C11	P1	Pd1	119.06(8)	C37	C36	C35	120.2(3)
C11	P1	C17	107.41(11)	C24	C23	P1	109.17(16)
C11	P1	C23	101.05(11)	C3	C4	N5	119.2(2)
F2	P4	F1	89.49(9)	C41	C40	C39	120.5(3)
F4	P4	F1	90.15(9)	C12	C13	C14	120.3(3)
F4	P4	F2	179.61(11)	C31	C32	P3	109.64(16)
F5	P4	F1	90.51(10)	C8	C9	C10	111.1(3)
F5	P4	F2	89.83(11)	C40	C41	C42	120.0(3)
F5	P4	F4	90.04(12)	C19	C20	C21	120.7(3)
F3	P4	F1	179.16(11)	C2	C3	C1[1]	121.4(2)
F3	P4	F2	90.23(10)	C4	C3	C1[1]	120.3(2)
F3	P4	F4	90.13(10)	C4	C3	C2	118.29(19)
F3	P4	F5	90.28(11)	C19	C18	C17	120.2(3)
F6	P4	F1	89.93(11)	C20	C21	C22	120.3(3)
F6	P4	F2	90.07(12)	C30	C29	C28	120.1(3)
F6	P4	F4	90.06(12)	C7	C6	C5	110.9(2)
F6	P4	F5	179.55(12)	C18	C19	C20	119.8(3)
F6	P4	F3	89.28(11)	C6	C7	C8	111.9(2)
C3[1]	C1	C2	120.9(2)	C37	C38	C33	120.2(3)
C10	C5	N5	115.7(2)	C16	C11	P1	117.66(18)
C6	C5	N5	110.02(19)	C12	C11	P1	122.4(2)
C6	C5	C10	110.9(2)	C12	C11	C16	119.3(2)
C5	N5	Pd1	129.12(14)	C43	C44	C39	120.1(3)
C4	N5	Pd1	110.08(15)	C36	C35	C34	119.7(3)

Table S6 Bond Angles for JM VILA 21VAA045. Compound 10 (from 2a).

Atom	Atom	Atom	Angle/˚	Atom	Atom	Atom	Angle/˚
C4	N5	C5	120.78(19)	C11	C16	C15	120.5(3)
C9	C10	C5	109.9(2)	C44	C43	C42	120.1(3)
C32	C31	P2	110.63(16)	C11	C12	C13	119.8(3)
C21	C22	C17	119.5(3)	C38	C37	C36	120.0(3)

[1]-X,1-Y,-Z

Table S7 Crystal data and structure refinement for 5b.

Identification code	JM VILA 21vaa008
Empirical formula	$C_{42}H_{38}F_6O_2P_4Pd$
Formula weight	919.069
Temperature/K	100.00
Crystal system	monoclinic
Space group	$P2_1/n$
a/Å	13.1305(4)
b/Å	14.8772(4)
c/Å	20.4043(7)
α/°	90
β/°	102.551(1)
γ/°	90
Volume/Å^3	3890.6(2)
Z	4
ρ_{calc}g/cm^3	1.569
μ/mm^{-1}	0.708
F(000)	1862.9
Crystal size/mm^3	0.09 × 0.04 × 0.03
Radiation	Mo Kα (λ = 0.71073)
2Θ range for data collection/°	4.92 to 52.74
Index ranges	-15 ≤ h ≤ 16, -18 ≤ k ≤ 18, -25 ≤ l ≤ 25
Reflections collected	59817
Independent reflections	7952 [R_{int} = 0.0481, R_{sigma} = 0.0303]
Data/restraints/parameters	7952/0/496
Goodness-of-fit on F^2	1.051
Final R indexes [I>=2σ (I)]	R_1 = 0.0407, wR_2 = 0.0891
Final R indexes [all data]	R_1 = 0.0501, wR_2 = 0.0937
Largest diff. peak/hole / e Å^{-3}	1.29/-0.77

Table S8 Bond Lengths for JM VILA 21vaa008. Compound 5b

Atom	Atom	Length/Å	Atom	Atom	Length/Å
Pd1	P1	2.3098(8)	C36	C35	1.385(5)
Pd1	P2	2.2762(8)	C36	C31	1.391(5)
Pd1	P3	2.3334(8)	C37	C38	1.396(5)
Pd1	C6	2.080(3)	C37	C42	1.384(5)
P1	C21	1.851(3)	C38	C39	1.389(5)
P1	C15	1.819(3)	C3	C8	1.472(5)
P1	C9	1.810(3)	C27	C26	1.386(5)
P2	C23	1.806(3)	C23	C24	1.395(5)
P2	C22	1.824(3)	C33	C32	1.376(5)
P2	C29	1.824(3)	C33	C34	1.390(6)
P4	F4	1.601(2)	C15	C20	1.392(5)
P4	F2	1.598(2)	C15	C16	1.397(5)
P4	F6	1.586(3)	C32	C31	1.402(5)
P4	F5	1.548(3)	C41	C42	1.390(5)
P4	F3	1.584(3)	C41	C40	1.376(6)
P4	F1	1.567(3)	C34	C35	1.384(5)
P3	C37	1.816(3)	C29	C30	1.542(4)
P3	C31	1.816(3)	C20	C19	1.386(5)
P3	C30	1.845(3)	C24	C25	1.388(5)
O2	C8	1.212(4)	C26	C25	1.379(5)
C6	C1	1.429(4)	C9	C14	1.393(4)
C6	C5	1.387(5)	C9	C10	1.385(5)
O1	C7	1.216(4)	C12	C13	1.380(5)
C2	C1	1.398(4)	C12	C11	1.377(5)
C2	C3	1.382(5)	C13	C14	1.389(5)
C1	C7	1.476(4)	C16	C17	1.384(5)
C21	C22	1.542(4)	C19	C18	1.389(6)
C4	C5	1.393(5)	C17	C18	1.385(6)
C4	C3	1.391(5)	C39	C40	1.370(5)
C28	C27	1.382(5)	C10	C11	1.385(5)
C28	C23	1.399(5)			

Table S9 Bond Angles for JM VILA 21vaa008. Compound 5b.

Atom	Atom	Atom	Angle/°	Atom	Atom	Atom	Angle/°
P2	Pd1	P1	83.97(3)	C23	C28	C27	119.7(3)
P3	Pd1	P1	163.65(3)	C31	C36	C35	120.7(3)
P3	Pd1	P2	83.48(3)	C38	C37	P3	117.0(3)
C6	Pd1	P1	94.63(8)	C42	C37	P3	124.0(3)
C6	Pd1	P2	176.84(9)	C42	C37	C38	118.9(3)
C6	Pd1	P3	97.36(8)	C39	C38	C37	120.3(3)
C21	P1	Pd1	107.92(10)	C4	C3	C2	118.7(3)
C15	P1	Pd1	114.87(10)	C8	C3	C2	121.3(3)
C15	P1	C21	105.10(15)	C8	C3	C4	120.1(3)
C9	P1	Pd1	118.94(11)	C26	C27	C28	120.9(3)
C9	P1	C21	104.33(15)	C28	C23	P2	120.4(3)
C9	P1	C15	104.40(15)	C24	C23	P2	120.3(3)
C23	P2	Pd1	115.56(11)	C24	C23	C28	119.3(3)
C22	P2	Pd1	109.00(11)	C34	C33	C32	120.3(3)
C22	P2	C23	105.86(15)	C20	C15	P1	121.3(3)
C29	P2	Pd1	108.61(11)	C16	C15	P1	119.9(3)
C29	P2	C23	105.00(15)	C16	C15	C20	118.7(3)
C29	P2	C22	112.89(15)	C31	C32	C33	120.4(3)
F2	P4	F4	178.59(15)	C40	C41	C42	120.4(4)
F6	P4	F4	90.11(16)	C3	C8	O2	124.6(3)
F6	P4	F2	90.87(15)	C35	C34	C33	119.7(4)
F5	P4	F4	88.47(15)	C41	C42	C37	120.1(4)
F5	P4	F2	90.58(15)	C21	C22	P2	105.7(2)
F5	P4	F6	177.5(2)	C30	C29	P2	104.7(2)
F3	P4	F4	89.92(15)	C19	C20	C15	120.8(3)
F3	P4	F2	91.15(15)	C34	C35	C36	120.0(4)
F3	P4	F6	86.6(2)	C36	C31	P3	121.4(3)
F3	P4	F5	91.3(2)	C32	C31	P3	119.9(3)
F1	P4	F4	89.66(14)	C32	C31	C36	118.7(3)
F1	P4	F2	89.33(14)	C25	C24	C23	120.0(3)
F1	P4	F6	89.6(2)	C25	C26	C27	119.4(4)
F1	P4	F5	92.5(3)	C1	C7	O1	123.9(3)
F1	P4	F3	176.2(2)	C14	C9	P1	120.8(2)
C37	P3	Pd1	119.57(11)	C10	C9	P1	120.0(2)
C31	P3	Pd1	114.96(11)	C10	C9	C14	119.2(3)
C31	P3	C37	105.11(15)	C26	C25	C24	120.6(3)
C30	P3	Pd1	107.07(10)	C11	C12	C13	120.2(3)
C30	P3	C37	105.78(15)	C29	C30	P3	109.1(2)
C30	P3	C31	102.75(15)	C14	C13	C12	120.1(3)
C1	C6	Pd1	119.7(2)	C17	C16	C15	120.3(3)
C5	C6	Pd1	124.3(2)	C18	C19	C20	120.1(4)
C5	C6	C1	116.0(3)	C13	C14	C9	119.9(3)
C3	C2	C1	120.6(3)	C18	C17	C16	120.6(4)
C2	C1	C6	121.5(3)	C40	C39	C38	120.2(3)
C7	C1	C6	121.1(3)	C39	C40	C41	120.0(3)
C7	C1	C2	117.4(3)	C11	C10	C9	120.7(3)
C22	C21	P1	111.9(2)	C17	C18	C19	119.5(3)
C3	C4	C5	120.9(3)	C10	C11	C12	119.8(3)
C4	C5	C6	122.3(3)				
